# Maca (*L. meyenii*) for improving sexual function: a systematic review

**DOI:** 10.1186/1472-6882-10-44

**Published:** 2010-08-06

**Authors:** Byung-Cheul Shin, Myeong Soo Lee, Eun Jin Yang, Hyun-Suk Lim, Edzard Ernst

**Affiliations:** 1 Division of Clinical Medicine, School of Oriental Medicine, Pusan National University, Yangsan, South Korea; 2 Division of Standard Research, Korea Institute of Oriental Medicine, Daejeon, South Korea; 3 Department of Nursing, Hanyang University Kuri Hospital, Kuri, Gyeonggi-Do, South Korea; 4 Complementary Medicine, Peninsula Medical School, University of Exeter, Exeter, UK

## Abstract

**Background:**

Maca (*Lepidium meyenii*) is an Andean plant of the brassica (mustard) family. Preparations from maca root have been reported to improve sexual function. The aim of this review was to assess the clinical evidence for or against the effectiveness of the maca plant as a treatment for sexual dysfunction.

**Methods:**

We searched 17 databases from their inception to April 2010 and included all randomised clinical trials (RCTs) of any type of maca compared to a placebo for the treatment of healthy people or human patients with sexual dysfunction. The risk of bias for each study was assessed using Cochrane criteria, and statistical pooling of data was performed where possible. The selection of studies, data extraction, and validations were performed independently by two authors. Discrepancies were resolved through discussion by the two authors.

**Results:**

Four RCTs met all the inclusion criteria. Two RCTs suggested a significant positive effect of maca on sexual dysfunction or sexual desire in healthy menopausal women or healthy adult men, respectively, while the other RCT failed to show any effects in healthy cyclists. The further RCT assessed the effects of maca in patients with erectile dysfunction using the International Index of Erectile Dysfunction-5 and showed significant effects.

**Conclusion:**

The results of our systematic review provide limited evidence for the effectiveness of maca in improving sexual function. However, the total number of trials, the total sample size, and the average methodological quality of the primary studies were too limited to draw firm conclusions. More rigorous studies are warranted.

## Background

Sexual problems (or sexual dysfunction) are widespread and adversely affect mood, well-being, and interpersonal relationships [1]. They occur in 20%-30% of men and 40-45% of women according to 18 descriptive epidemiological studies from around the world [2]. Most sexual problems relate to sexual desire (interest in sex) in both females and males and male erectile dysfunction (ED) [2]. Current pharmacological interventions for the management of sexual problems include oral drugs, intrapenile therapies (intra-urethral suppositories and intracavernous injections) and penile prosthesis implantation for males and hormonal therapy for females. Although considerable advances have been made, the ideal treatment for ED has not been identified. The treatment for sexual problems in females is also problematic [3]. Furthermore, pharmacological treatments have been shown to result in several adverse effects, including risk of cancer, headache, rhinitis and dyspepsia [4-6]. Non-pharmacological treatments of female sexual problems includes vaginal electromyography biofeedback, pelvic floor physical therapy, (group) cognitive behavioural therapy, transcutaneous electrical nerve stimulation, and vestibulectomy [7]. Herbal therapies for ED or sexual dysfunction in males and females include yohimbine (*Pausinvstalia vohimbe*), which is burdened with serious adverse effects [8-10], ginkgo (*Ginkgo biloba*) and red ginseng (*Panax ginseng*) [10,11]. Several other botanical therapies for sexual dysfunction have also been introduced [8,10,12]. These are also often used for improving sexual function in healthy subjects.

Maca (*Lepidium meyenii*) is an Andean plant that belongs to the brassica (mustard) family. Maca has been used for centuries in the Andes to enhance fertility in humans and animals [12,13]. Preparations from the maca root have been reported to improve sexual function in healthy populations [13]. Although maca is a plant extract and not a drug, it is one of the most commonly cited "natural drugs" on the Internet for the improvement of sexual desire. The hypothesis that maca may be effective in improving sexual function is supported by several lines of evidence. Animal experiments suggest that maca has spermatogenic and fertility-enhancing activities, which are likely due to the phytosterols or phytoestrogens present in the maca [14]. Several in vivo studies have shown that maca may improve sexual behaviour and enhance androgen-like effects in rats [15,16]. Recent clinical trials have also suggested significant effects of maca for increasing sperm count and mobility and improving sexual function in humans [17,18]. The potential bioactive ingredients in maca include macaridine, macamides, macaene, gluosinolates, maca alkaloid, and maca nutrients [14]. However, these data are insufficient for determining whether maca is clinically effective. Currently, no systematic review of this subject is available. The aims of this systematic review are to summarise and critically assess the evidence from randomised clinical trials (RCTs) for or against the effectiveness of maca in the improvement of sexual function, including sexual desire and sexual responses.

## Methods

### Data sources

The following electronic databases were searched from inception through April 2010: Medline, AMED, CINAHL, EMBASE, PsycInfo, the Cochrane Central Register of Controlled Trials and the Cochrane Database of Systematic Review, DARE, Psychology and Behavioral Sciences Collection, six Korean Medical Databases (Korean Studies Information, DBPIA, Korea Institute of Science and Technology Information, KERIS, KoreaMed, and Korean National Assembly Library), Chinese Medical Databases (CNKI; http://www.cnki.net), and The Japanese Science and Technology Information Aggregator, Electronic. The search terms used were "*Lepidium meyenii" *AND sexual dysfunction OR erectile dysfunction OR sexual function). The search strategy was composed of a mixture of free text and thesaurus terms [see Additional file 1]. We also manually searched our departmental files and relevant journals (Focus on Alternative and Complementary Therapies and Forschende Komplementärmedizin und Klassische Naturheilkunde) until April 2010. The references in all located articles were manually searched for further relevant articles. Dissertations and abstracts were included.

### Study selection

Trials involving people with normal sexual function and those with sexual dysfunction were included. All articles that reported an RCT in which humans were treated with any type of maca *(Lepidium meyenii*) preparation, regardless of origin, were included. Trials were included if they employed maca as the sole treatment or as an adjunct to conventional treatments compared to a placebo control. Studies that used at least one measure related to sexual function were included. We excluded trials comparing two different types or dosages of maca and those in which no clinical data or insufficient data for comparison were reported. For duplicate publications with different outcome measures originating from one trial published as separate papers, the original publication was given priority, and all others were excluded. No language restrictions were imposed.

### Extraction of data and assessment of risk of bias

All of the included articles were read in full. Three independent reviewers (BCS, MSL, and EJY) extracted the data, including methods (e.g., design, blinding, duration of follow-up), sample (e.g., population size, conditions, age, duration of disease), intervention and control treatment, and outcome measures, according to predetermined criteria (Table 1). The Cochrane classification (i.e., sequence generation, blinding, incomplete outcome measures, and allocation concealment) was applied to evaluate the risk of bias [19]. Differences in opinions between the reviewers were settled through discussion.

### Data Synthesis

We had originally intended to conduct a formal meta-analysis. However, statistical and clinical heterogeneity prevented us from doing so. The main ways we would have done were like followings. The post-treatment values (the end of treatment) of the outcome measures were used to assess differences between the intervention groups and the control groups. We did not include the follow-up treatment values. Standardised mean differences (SMDs) were used for pooling the outcomes related with sexual function with a random effects model. SMDs and 95% confidence intervals (CIs) were calculated using Cochrane Collaboration's software (Review Manager Version 5.0 for Windows, Copenhagen: The Nordic Cochrane Centre). The mean difference (MD) for each outcome measure was calculated using the same software. Statistical heterogeneity was evaluated using a χ^2 ^test and I^2 ^statistics (low = 25%; moderate = 50%; high = 75%).

## Results

### Study description

**Figure 1:**
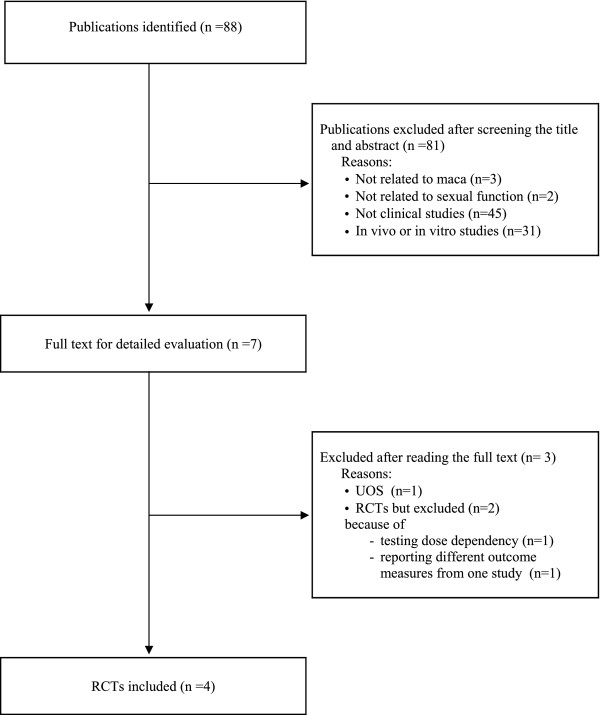
**Flow chart for the selection of included trials**. RCT: randomised clinical trial; UOS: uncontrolled observational study.

The literature searches revealed 88 articles, of which 84 had to be excluded (Figure 1). Among these, one RCT was excluded because it compared two different dosages [20] and another because it reported different outcome measures from one trial [18]. One trial was excluded because of the absence of a control group [17]. Four RCTs met our inclusion criteria, and their key data are summarised in Table 1[21-24]. Of the four studies, one RCT was conducted in Italy [21], one in Peru [23], one in Australia [22], and one in the UK [24]. One RCT adopted a two-armed parallel group design [21], one employed a three-armed parallel group design [23], and the other two used a crossover design [22,24]. The four trials included a total of 131 subjects. Two RCTs contributed most to the sample (n = 50, and 57) [21,23], while the other two RCTs were relatively small (n = 8, and 16) [22,24]. Two RCTs [21,22] employed dried maca, and the other two [23,24] used gelatinised maca. All of the participants in the four studies ingested the maca orally. Dosages were 1.5 g to 3.5 g of maca daily for 2 or 12 weeks. The age range of male participants was from 21 to 56 years for healthy subjects and 36 ± 5 years for patients with ED, while the age range of postmenopausal women was 54 ± 11 years. The outcome measures used in these trials included the International Index of Erectile Dysfunction (IIEF-5) [21], the sexual dysfunction Green Climacteric Scale [22], sexual desire according to the 6-point Likert scale [23], and the Sexual Desire Inventory [24]. Three RCTs [22-24] used commercial products, and one RCT [21] tested natural dried maca.

**Table 1:** Summary of randomised clinical trials with maca for sexual function

First author (year) location	Sample size/condition Age (years) Sex (M/F) Duration of disease	Intervention (regimen)	Control intervention (regimen)	Main outcome measures	Results	Adverse effects
Zenico (2009) [21] Italy	50 mild ED 36 (SDs, 5) (50/0) n.r.	(A) Maca (pulverised dehydrated maca roots directly imported from Peruvian Andes, tablets, 2400 mg/d, 1200 mg/d, 2 daily for 12 weeks, n = 25), no follow-up	(B) Placebo tablets (2400 mg/d, 1200 mg/d, 2 daily for 12 weeks, n = 25)	IIEF-5	Intergroup: MD, 1.10 [0.61, 1.59], P < 0.001	n.r.
					Within group: (A) P < 0.05, (B) P < 0.05	
Brooks (2003) [22] Australia	16 healthy postmenopausal women with moderate severity of menopausal symptoms	(A) Maca (company commercial product, dried maca powder, 3500 mg/d, daily for 6 weeks, n = 14), 6 weeks follow-up	(B) Placebo (refined white rice flour, 3500 mg/d, daily for 6 weeks, n = 14)	Sexual dysfunction (GCS)	Intergroup: MD, 0.70 [0.08, 1.32], P < 0.05	n.r.
	54 (SDs, 11) (0/16) >12 months amenorrhea				Within group: (A) P < 0.05, (B) NS	
Gonzales (2002) [23] Peru	57 adult healthy men 21-56 (57/0) N/A	(A) Maca (company commercial product, gelatinised maca, 1500 mg/d; 500 mg/d, 3 tablets, daily for 12 weeks, n = 30), no follow-up	(C) Placebo tablets (n.r., daily for 12 weeks, n = 12)	Self-perception on sexual desire	Intergroup: MD, 0.51 [-0.35, 1.37], NS at 4 weeks; MD, 1.64 [1.07, 2.21], P < 0.008 at 8 weeks; MD, 1.64 [1.07, 2.21], P < 0.006 at 12 weeks	n.r.
		(B) Maca (company commercial product, gelatinised maca, 3000 mg/d; 500 mg/d, 6 tablets, daily for 12 weeks, n = 15), no follow-up			Within group: (A) P < 0.05 at 4, 8, and 12 weeks, (B) NS at 4, 8, and 12 weeks	
Stone (2009) [24] UK	8 experienced and endurance trained male (cyclists)	(A) Maca (company commercial product, gelatinised maca, 2000 mg/d for 2 weeks, n = 8), no follow-up	(B) Placebo capsules (Arabic gum, n = 8)	Sexual Desire Inventory	Intergroup: MD, 6.38 [-11.32, 24.08], NS	n.r.
	30 (SDs, 7) 8/0 N/A Cross-over (1 week washout period)				Within group: (A) P = 0.01, (B) P = 0.90	

ED: erectile dysfunction; GCS: Greene Climacteric Scale; IIEF-5: International Index of Erectile Dysfunction; NS: not significant; n.r.: not reported; SDs: standard deviations

### Risk of bias

None of the included RCTs reported their methods of sequence generation. All of the included trials employed a double-blind design. One trial reported complete outcome measures [22]. None employed allocation concealment.

### Outcomes

#### Patients with sexual dysfunction

One RCT compared the effects of maca vs. placebo treatments in patients with erectile dysfunction. This trial [21] showed positive effects of maca on the IIEF-5 in patients with mild ED compared to the placebo control (MD, 1.10, 95% CIs, 0.61 to 1.59, P < 0.001) [21].

#### Healthy volunteers

Three RCTs tested the effects of maca vs. placebo on sexual function in healthy postmenopausal women [22], healthy adult men [23] or male cyclists [24]. One RCT [22] reported positive effects of maca on sexual function in healthy menopausal women compared with the placebo control (MD, 0.70, 95% CIs, 0.08 to 1.32, P < 0.05). The other RCT [23] tested both a high dosage of maca (3 g/d) and a low dosage of maca (1.5 g/d) on sexual desire compared to a placebo control and reported positive effects of both dosages of maca after 8 weeks (MD, 1.64, 95% CIs, 1.07 to 2.21, P < 0.01) and 12 weeks (MD, 1.64, 95% CIs, 1.07 to 2.21, P < 0.01). The further RCT [24], which had a very small sample size, failed to show positive effects of maca in the improvement of sexual desire (MD, 6.38, 95% CIs, -11.32 to 24.08, NS).

### Adverse effects

None of included trials attempted to assess the adverse effects of maca.

## Discussion

Few RCTs have tested the effects of maca on sexual function. This review found limited evidence from four small trials that suggested that maca is effective in improving sexual desire after at least 6 weeks. However, this finding is limited because the studies were underpowered. In particular, two studies found no effect after 2 or 4 weeks of treatment. Evidence from other studies suggests that maca may be effective for sexual dysfunction in patients with ED and postmenopausal women after 12 weeks or 6 weeks, respectively. However, the total number of trials, the total sample size, and the average risk of bias in the primary studies were too limited to draw firm conclusions.

All of the RCTs employed double-blind methods, but none of the included trials reported methods of sequence generation for randomisation and allocation concealment. Trials with inadequate sequence generation and inadequate allocation concealment are likely to show exaggerated treatment effects [25] and thus limit the reliability of the study results. Although all included RCTs used placebo controls, none reported the success of blinding. None of the studies reported a power calculation, and sample sizes were very small in some of the RCTs (ranging from 8 to 57). One article has a poor description of the outcome and was therefore difficult to interpret [23]. In this trial, only the mean average of two active groups (high and low dosage of maca) was compared with the control group. One RCT failed to include washout periods between crossover periods [22]. One of the main problems in crossover trials is the possibility of a carry-over effect. Therefore, this RCT is difficult to interpret. The other RCT has a small sample size and is therefore susceptible to a type II error [24].

Four kinds of questionnaires were used for measuring sexual function or sexual desire, including the IIEF-5 [21], Green Climacteric Scale (GCS) [22], subjective Likert scale [23], and Sexual Desire Inventory [24]. Three RCTs [21,22,24] employed validated questionnaires, while one RCT [23] did not. Because the GCS is not specified for sexual function, it might not be sensitive enough to detect changes in sexual dysfunction. One trial tested changes in sexual desire compared to a baseline value with a 6-point Likert scale. It is not clear whether the authors used validated scales. It is important that only validated questionnaires are employed; otherwise, the outcome measures used have less established reliability and validity, data derived from them are subject to bias, and comparisons between the results of different studies are problematic.

The extent to which the therapeutic effects of maca depend on the type of maca used and the amount of various constituents in the preparation is unclear. The optimum dose of maca is unknown. Single-dose studies used extract quantities ranging from 1.5 g/d to 3.0 g/d. One excluded RCT compared two dosages of maca, 1.5 g/d and 3.0 g/d, for the management of SSRI-induced sexual dysfunction, but the results of that study failed to show differences between the two doses [20]. Further studies are required to identify the optimal dose.

One argument for the use of maca for the management of sexual function might be that it causes fewer adverse effects than conventional drug treatments. However, none of the four RCTs described here assessed the adverse effects of maca. This should be tested in future studies.

One could question the validity of our conclusions by pointing to the review method used (reviewing a small number of trials with many limitations). However, the reasons for doing a systematic review include answering questions not addressed by individual studies, settling controversies arising from apparently conflicting studies, and generating new hypotheses [19].

The limitations of our systematic review pertain to the paucity of data and the potential incompleteness of the evidence reviewed. None of the three RCTs have been submitted for independent replication. We aimed to identify all studies on the topic. The distorting effects of publication bias and location bias on systematic reviews and meta-analyses are well documented [26]. None of the RCTs included in our review were fully successful in minimising bias. Collectively, these facts seriously limit the conclusiveness of our systematic review.

Our decision to exclude non-randomised trials might also be criticised. However, we strongly feel that non-randomisation introduces a selection bias that, in turn, would render any results uninterpreptible. The exclusion of RCTs comparing different dosages might be criticised. We feel that such trials would not give objective clinical information of value. Moreover, these studies cannot provide reliable data on the effectiveness of maca. Therefore, we believe the exclusion of such studies was the correct decision.

Regarding an implication for practice, this review identifies limited evidence for the use of maca in improving sexual function in healthy subjects or patients with ED. However, practitioners and clinical decision makers need to be aware that there are too many limitations to draw firm conclusion. Future trials testing the effects of maca should adhere to rigorous trial designs that adequately suit the research question being asked. Such trials should preferably be randomised, control for placebo effects, be double-blinded, adequately conceal allocation, have optimal treatment dosages and sample sizes based on proper sample size calculations, use validated outcome measures, assess other outcomes such as quality of life or partner outcome as well as adverse effects, and include a full description of the actual interventions being tested.

## Conclusion

The results of our systematic review provide limited evidence for the effectiveness of maca in the improvement of sexual function. However, the total number of trials, the total sample size, and the average methodological quality of the primary studies were too limited to draw firm conclusions. Moreover, our current knowledge of the risks of maca intake is insufficient. More rigorous studies are warranted.

## Competing interests

The authors declare that they have no competing interests.

## Authors' contributions

BCS and MSL designed the review, performed searches, appraised and selected trials, extracted data, contacted authors for additional data, carried out analyses and interpretations of the data, and drafted this report. EJY reviewed and critiqued this review and report and assisted with interpretation of the data. HSL and EE reviewed and critiqued the review protocol and this report and assisted in designing the review. All authors read and approved the final manuscript.

## Pre-publication history

The pre-publication history for this paper can be accessed here:

http://www.biomedcentral.com/1472-6882/10/44/prepub

## Supplementary Material

Additional file 1 **The search strategies for MEDLINE**. The data provided the details of search strategies for MEDLINE. Click here for file
