# Supplementary material for: Maca (L. meyenii) for improving sexual function: a systematic review
Source: BMC Complement Altern Med. 2010 Aug 6;10:44. doi: 10.1186/1472-6882-10-44 (PMC2928177; doi:10.1186/1472-6882-10-44)
Supplement: Additional file 1 — The search strategies for MEDLINE. The data provided the details of search strategies for MEDLINE. [file 1472-6882-10-44-S1.DOC]

**Additional file 1.** The search strategies for MEDLINE

1. exp Lepidium meyenii/

2. (lepidium OR lepidiums OR lepidium meyenii OR lepidium meyenius OR meyenii, lepidium OR meyenius, lepidium OR maca OR macas).tw

3. exp Erectile Dysfunction/

4. exp Sexual Dysfunction, Physiological/

5. exp Sexual function/

6. (sexual dysfunction or impotence or lubrication or coitus or copulation) .tw

7. (sex* adj (dysfunct* or satisf* or problem* or symptom* or arousal* or activit*)).tw

8. ([orgasm or libido or lubricat* or dyspareunia or “hypoactive sexual desire disorder” or “sexual aversion or coition”)](javascript:__doPostBack('ctl00$ctl00$BaseFindField$FindField$ctl00$SpellCheckerControl$rpSuggest$ctl00$btnSuggestTerm','')). tw

9. (“sexual intercourse” or “erectile dysfunction” or erect* or “sexual attraction” or intimacy or procreat* or relations or sex or sex act or “sexual congress” or “sexual relation” or arousal or “penile erection” or “vaginal dryness” or “sexual pain” OR vaginismus).tw

10. (pain* adj intercourse).tw

11. (sex* adj pain*).tw

12. (ejaculation dysfunction or premature ejaculation or early ejaculation or delayed ejaculation or “retarded ejaculation” or anejaculation or “painful ejaculation” or “retrograde ejaculation” or “anterograde ejaculation” or “inhibited ejaculation”).tw

13. 1 or 2

14. 3 or 4 or 5 or 6 or 7 or 8 or 9 or 10 or 11 or 12

15. 13 and 14
